# The PERSonalized Glucose Optimization Through Nutritional Intervention (PERSON) Study: Rationale, Design and Preliminary Screening Results

**DOI:** 10.3389/fnut.2021.694568

**Published:** 2021-06-30

**Authors:** Anouk Gijbels, Inez Trouwborst, Kelly M. Jardon, Gabby B. Hul, Els Siebelink, Suzanne M. Bowser, Dilemin Yildiz, Lisa Wanders, Balázs Erdos, Dick H. J. Thijssen, Edith J. M. Feskens, Gijs H. Goossens, Lydia A. Afman, Ellen E. Blaak

**Affiliations:** ^1^Division of Human Nutrition and Health, Wageningen University, Wageningen, Netherlands; ^2^Top Institute Food and Nutrition, Wageningen, Netherlands; ^3^Department of Human Biology, Maastricht University Medical Center+, Maastricht, Netherlands; ^4^Department of Physiology, Radboud Institute for Health Sciences, Radboud University Medical Center, Nijmegen, Netherlands; ^5^Maastricht Centre for Systems Biology, Maastricht University, Maastricht, Netherlands; ^6^Research Institute for Sport and Exercise Sciences, Liverpool John Moores University, Liverpool, United Kingdom

**Keywords:** precision nutrition, personalized nutrition, insulin resistance, metabolic phenotype, glucose homeostasis, obesity, dietary intervention study, randomized clinical trial

## Abstract

**Background:** It is well-established that the etiology of type 2 diabetes differs between individuals. Insulin resistance (IR) may develop in different tissues, but the severity of IR may differ in key metabolic organs such as the liver and skeletal muscle. Recent evidence suggests that these distinct tissue-specific IR phenotypes may also respond differentially to dietary macronutrient composition with respect to improvements in glucose metabolism.

**Objective:** The main objective of the PERSON study is to investigate the effects of an optimal vs. suboptimal dietary macronutrient intervention according to tissue-specific IR phenotype on glucose metabolism and other health outcomes.

**Methods:** In total, 240 overweight/obese (BMI 25 – 40 kg/m^2^) men and women (age 40 – 75 years) with either skeletal muscle insulin resistance (MIR) or liver insulin resistance (LIR) will participate in a two-center, randomized, double-blind, parallel, 12-week dietary intervention study. At screening, participants undergo a 7-point oral glucose tolerance test (OGTT) to determine the hepatic insulin resistance index (HIRI) and muscle insulin sensitivity index (MISI), classifying each participant as either “No MIR/LIR,” “MIR,” “LIR,” or “combined MIR/LIR.” Individuals with MIR or LIR are randomized to follow one of two isocaloric diets varying in macronutrient content and quality, that is hypothesized to be either an optimal or suboptimal diet, depending on their tissue-specific IR phenotype (MIR/LIR). Extensive measurements in a controlled laboratory setting as well as phenotyping in daily life are performed before and after the intervention. The primary study outcome is the difference in change in disposition index, which is the product of insulin sensitivity and first-phase insulin secretion, between participants who received their hypothesized optimal or suboptimal diet.

**Discussion:** The PERSON study is one of the first randomized clinical trials in the field of precision nutrition to test effects of a more personalized dietary intervention based on IR phenotype. The results of the PERSON study will contribute knowledge on the effectiveness of targeted nutritional strategies to the emerging field of precision nutrition, and improve our understanding of the complex pathophysiology of whole body and tissue-specific IR.

**Clinical Trial Registration:** https://clinicaltrials.gov/ct2/show/NCT03708419, clinicaltrials.gov as NCT03708419.

## Introduction

The prevalence of overweight and related metabolic disturbances, including impaired glucose homeostasis, is rising at an alarming rate, thereby increasing the risk for type 2 diabetes mellitus (T2DM) and cardiovascular disease (CVD) (1). Dietary modulation can effectively lower blood glucose levels and reduce the risk of chronic metabolic diseases, independent of weight loss (2, 3). Interestingly, there is great heterogeneity in individuals' metabolic response to dietary interventions (4, 5). Part of this heterogeneity may be attributed to differences in adherence, but recent findings of large inter-individual variation in postprandial responses to standardized meals indicate that individuals actually respond differently to food (6, 7). This inter-individual variation in response to food has complex underpinnings that include biological (including genetic), environmental, and lifestyle factors, and may partly explain the differential metabolic impact of dietary interventions (4–9).

Whole-body insulin resistance (IR) reflects defective insulin action in tissues such as skeletal muscle, liver, adipose tissue, gut and brain, and is a major risk factor for T2DM and CVD. IR can develop concurrently in different tissues, but the severity of IR may vary between tissues (10, 11). Individuals may, for example, have IR predominantly in the liver or skeletal muscle (10). Liver insulin resistance (LIR) is manifested by impaired insulin-mediated suppression of hepatic glucose production (HGP), while muscle insulin resistance (MIR) is characterized by decreased insulin-mediated glucose disposal (11). The gold-standard method to quantify LIR and MIR is the two-step hyperinsulinemic-euglycemic clamp (11). Tissue-specific IR can also be modeled based on glucose and insulin responses during an oral glucose tolerance test (OGTT), which has been validated against the clamp technique (10, 12).

These tissue-specific IR phenotypes have previously been linked to distinct metabolic profiles, representing different etiologies toward T2DM and CVD (11, 13–15). More specifically, greater disturbances in circulating lipidome (13) and metabolome profiles (14) have been found in individuals with more pronounced LIR as compared to individuals with more pronounced MIR. Additionally, in individuals with LIR, abdominal subcutaneous adipose tissue (scAT) has been characterized by higher expression of genes related to extracellular modeling, whilst MIR has been associated with higher expression of genes related to inflammation in scAT, as well as higher levels of circulating plasma markers of systemic low-grade inflammation (16).

Recent findings indicate that these distinct metabolic phenotypes may respond differently to dietary macronutrient manipulation with regard to outcomes of glucose homeostasis, ectopic fat deposition, and tissue-specific lipid metabolism amongst others (15, 17). Indeed, a *post-hoc* analysis of the CORDIOPREV-DIAB study has indicated that a low-fat, high-complex carbohydrate diet may be particularly beneficial with respect to improvement in glucose metabolism for individuals with predominant LIR, while individuals with predominant MIR seem to benefit more from a Mediterranean diet high in monounsaturated fatty acids (MUFA) (18). Therefore, further characterization of these IR phenotypes as well as studying these metabolic phenotypes in relation to dietary intervention outcomes may be a promising strategy to develop more personalized dietary interventions. In addition, improvement of glycemic control by more personalized dietary interventions may enhance mood, self-control, and cognitive function (1, 19–21). Such short-term benefits may in turn increase adherence to a healthy diet.

Importantly, prospective randomized controlled trials with a pre-specified hypothesis on differential metabolic responses to diets based on (metabolic) phenotype are largely lacking in the emerging field of precision nutrition. The PERSonalized glucose Optimization through Nutritional intervention (PERSON) study was designed to investigate the effects of an optimal compared to a suboptimal dietary intervention according to tissue-specific IR phenotype on glucose metabolism and other metabolic health outcomes. This two-center, 12-week dietary intervention study with a randomized, double-blind, parallel design, aims to enroll a total of 240 individuals with either LIR or MIR. Individuals are randomized to follow one of two diets that are hypothesized to target one of the two tissue-specific IR phenotypes.

Before and after the 12-week dietary intervention, individuals are extensively phenotyped both in laboratory settings and in daily life. The extensive phenotyping performed in this unique clinical trial allows for a comprehensive study of both the complex metabolic and lifestyle determinants of glucose homeostasis, as well as the dietary intervention effects on metabolic health and its metabolic underpinnings. In the present article, we describe the study design and measurements in detail, and present preliminary results of the screening population.

## Methods

### Study Design

**Figure 1:**
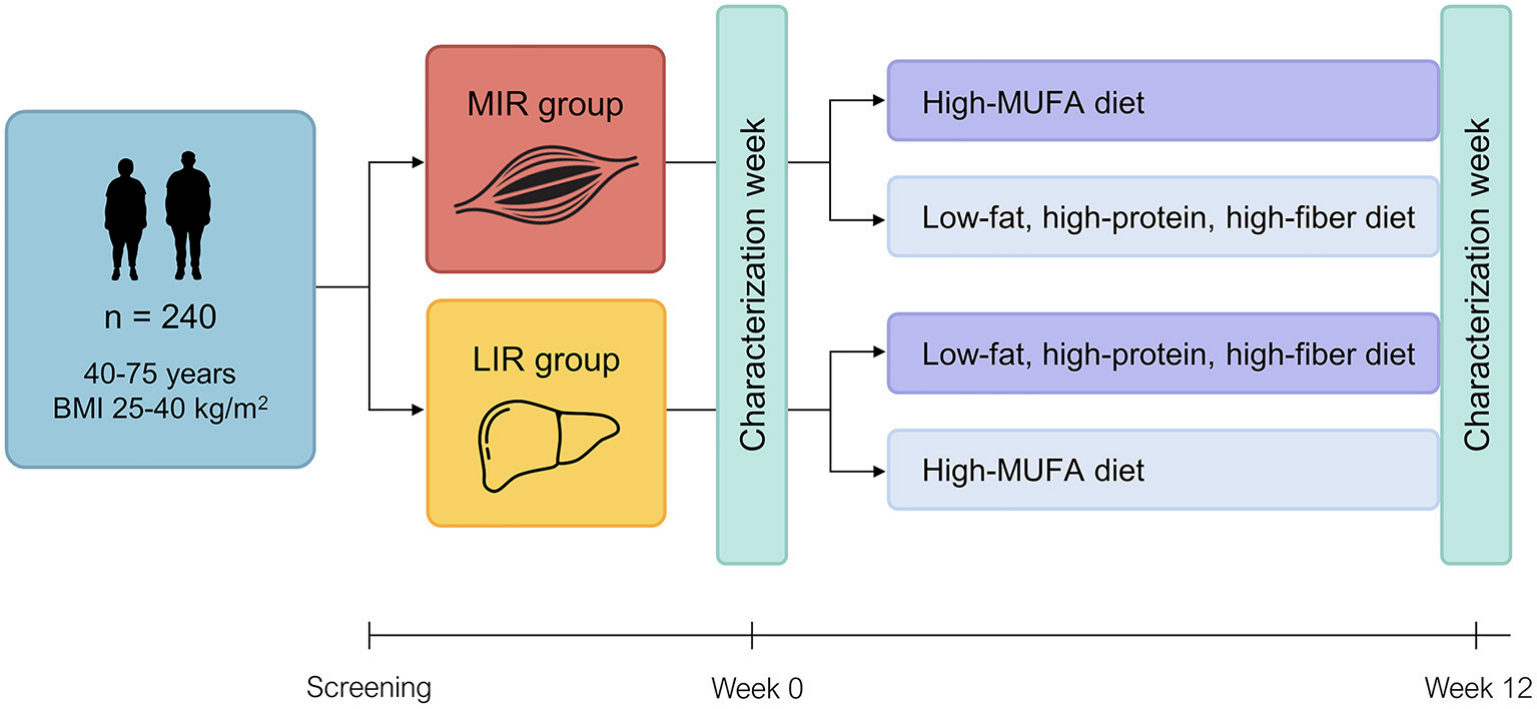
Study design of the PERSON study. Tissue-specific insulin resistance (MIR, muscle insulin resistance; LIR, liver insulin resistance) is assessed at screening using a 7-point oral glucose tolerance test and eligible participants with MIR or LIR are randomized to follow either their hypothesized optimal (dark purple) or suboptimal (light purple) diet for 12 weeks. Before and after the intervention, participants are extensively phenotyped during a “characterization week” in a controlled laboratory setting as well as in daily life. BMI, body mass index; MUFA, monounsaturated fatty acid.

The PERSON study is a two-center 12-week dietary intervention study with a randomized, double-blind, parallel design, carried out at Maastricht University Medical Center+ (MUMC+) and Wageningen University & Research (WUR), the Netherlands (Figure 1). The protocol was approved by the Medical Ethics Committee of MUMC+ (NL63768.068.17) and registered at ClinicalTrials.gov (identifier NCT03708419). The study is conducted according to the principles of the Declaration of Helsinki (revised version, 2013, Fortaleza, Brazil), and all subjects provide written informed consent before the start of the study.

The primary study outcome is the difference in change in disposition index, which is the product of insulin sensitivity and first-phase insulin secretion, between participants who received their hypothesized optimal or suboptimal diet. Secondary outcome parameters include whole-body and tissue-specific insulin sensitivity and glucose homeostasis, fasting and postprandial metabolic profile, vascular health, fecal microbiota composition and functionality, body fat distribution, ectopic fat accumulation, adipose tissue morphology and gene expression, skeletal muscle protein and gene expression, fasting immune metabolism, cognitive performance, and perceived well-being.

### Study Participants

From May 2018 onwards, subjects have been recruited via a volunteer database, flyers, and advertisements in local and online media. Inclusion criteria are age 40–75 years, body mass index (BMI) 25–40 kg/m^2^, body weight stability for at least 3 months (no weight gain or loss >3 kg), and tissue-specific IR, characterized as predominant LIR or MIR, as assessed by a 7-point OGTT (see “Screening”). Exclusion criteria include among others pre-diagnosis of T2DM, diseases or use of medication that affect glucose and/or lipid metabolism, major gastrointestinal diseases, history of major abdominal surgery, uncontrolled hypertension, smoking, alcohol consumption >14 units/wk, and >4 h/wk moderate-to-vigorous physical activity (see Supplementary Table 1 for the extensive list of exclusion criteria).

### Screening

Eligibility is assessed during a screening visit. Subjects are asked to refrain from alcohol and vigorous physical activity 24 h prior to the visit and arrive in the morning after a >10 h overnight fast. Body weight and height are measured in duplicate without shoes and heavy clothing to the nearest 0.1 kg and 0.1 cm, respectively. Waist and hip circumference are measured in duplicate to the nearest 0.1 cm using a non-flexible measuring tape. Blood pressure is measured in triplicate on the non-dominant arm with an automated sphygmomanometer after a 5-min rest with the subject in a supine position. The first measurement is used to acclimatize the subject to the measurements, and therefore omitted from the data.

Tissue-specific insulin resistance is assessed based on the glucose and insulin responses during a 7-point OGTT. Subjects ingest 200 ml of a ready-to-use 75 g glucose solution (Novolab) within 5 min, and blood samples are collected from the antecubital vein via an intravenous cannula under fasting conditions (*t* = 0 min) and after ingestion of the glucose drink (*t* = 15, 30, 45, 60, 90, and 120 min) for determination of plasma glucose and insulin concentrations. Hepatic IR and muscle insulin sensitivity are estimated using the calculations of Abdul-Ghani and colleagues (10). We have recently optimized the MISI calculator using the cubic spline method (12). The hepatic IR index (HIRI) and muscle insulin sensitivity index (MISI) are calculated according to the following formulas:


$$\begin{array}{l} \text{HIRI}=\text{glucose }0-30\;\left[AUC\;in\;mmol/L*h\right]\times insulin\;0-30\\ \;[AUC\;in\;pmol/L*h\;]\\ \text{MISI}=\frac{\text{dGlucose}/\text{dt}}{\text{insulin }\left[mean\;during\;OGTT\;in\;pmol/L\;\right]} \end{array}$$

In the formula for MISI, dG/dt is the rate of decay of plasma glucose concentration (mmol/L) during the OGTT, calculated as the slope of the least square fit to the decline in plasma glucose concentration from peak to nadir (10).

Glucose curves that are flagged by the calculator, because MISI calculation is not possible or possibly not biologically meaningful due to either a peak at 120 min, a “flat” curve, or non-negligible rebound (12), are visually inspected for classification of MIR and LIR. Both indices were developed and validated against gold standard measurements of tissue-specific IR by a hyperinsulinemic-euglycemic clamp (10, 12). To obtain study groups that are predominant LIR or MIR, subjects are classified as “No MIR/LIR,” “MIR,” “LIR,” or “combined MIR/LIR,” using tertile cutoffs for MISI and HIRI. The lowest tertile of MISI represents individuals with MIR, while the highest tertile of HIRI represents individuals with LIR. The cutoffs for these tertiles are based on values of a selected study population of The Maastricht Study (DMS) (22), which resembles the target population of the PERSON study. Since the prevalence of LIR seems lower in the PERSON study as compared to DMS after inclusion of *n* = 163 individuals, the median HIRI value in the PERSON study population will be used for classification of individuals that will be recruited for the remainder of the study.

From the OGTT, incremental area under the curve (iAUC) is calculated for both glucose and insulin using GraphPad Prism software (version 5.04). Only values above the fasting value are included in the iAUC. The homeostasis model assessment of insulin resistance (HOMA-IR) is calculated as (fasting glucose [mmol/L] × fasting insulin [mU/L])/22.5 (23). HOMA of β-cell function (HOMA- β) is calculated as (20 × fasting insulin [mU/L])/(fasting glucose [mmol/L] – 3.5). Matsuda index is defined as: [10,000 ÷ square root of [fasting plasma glucose (mmol/l) × fasting insulin (pmol/l)] × [mean glucose (mmol/l) x mean insulin (pmol/l)]], using glucose and insulin values of time points 0, 30, 60, 90, and 120 min (24). Disposition index is calculated as: [Matsuda index ^*^ (AUC30 min insulin/AUC30 min glucose)], where AUC30 min is the area under the curve between baseline and 30 min of the OGTT for insulin (pmol/l) and glucose (mmol/l) as calculated using the trapezoidal method, respectively. Glucose status is defined according to WHO criteria (25): normal glucose tolerance (NGT), fasting glucose <5.6 mmol/L and 120-min glucose <7.8 mmol/L; impaired fasting glucose (IFG), fasting glucose 5.6 – 6.9 mmol/L and 120-min glucose <7.8 mmol/L; impaired glucose tolerance (IGT), fasting glucose <5.6 mmol/L and 120-min glucose 7.8 – 11.0 mmol/L; combined IFG/IGT, fasting glucose 5.6 – 6.9 mmol/L and 120-min glucose 7.8-11.0 mmol/L; T2DM, fasting glucose ≥7.0 mmol/L and/or 120-min glucose ≥11.1 mmol/L.

Hb and the parameters of hepatic and renal function alanine aminotransferase (ALT), aspartate aminotransferase (AST), and creatinine are determined in fasting blood samples by the hospital laboratories of MUMC+ and Ziekenhuis Gelderse Vallei, Ede, the Netherlands. Habitual dietary intake is estimated by a validated 163-item semiquantitative food frequency questionnaire (FFQ) (26). Dietary misreporting is evaluated by Goldberg's method, using the ratio of daily energy intake (EI) to estimated basal metabolic rate (BMR) (27, 28). Energy under- (EI/BMR < 0.87) and overreporters (EI/BMR > 2.75) are excluded from data analyses. Data on demographics, medical history, family history of DM (≥1 first-degree relative with DM), medication use and lifestyle are collected by questionnaire. Education level is categorized into low (no education, primary education, lower or preparatory vocational education, lower general secondary education), medium (intermediate vocational education, higher general senior secondary education or pre-university secondary education) and high (higher vocational education, university). Perceived chronic stress is assessed with the Long-term Difficulties Inventory (29) and mental well-being with the RAND 36-Item Short Form Health Survey (RAND-36) (30) and the Social Production Function Instrument for the Level of Well-being (31).

### Randomization Procedure

Eligible subjects are randomly allocated to either their hypothesized optimal or suboptimal diet by an independent analyst using center-specific minimization (32, 33) with randomization factors of 1.0 for the LIR/MIR phenotype, and 0.8 for age and sex, and a base probability of 0.7 by means of biased-coin (34). Both researchers and participants are blinded to the participants' metabolic phenotype, and thus blinded to whether participants are allocated to their hypothesized optimal or suboptimal diet. Participants start the study within 3 months of the screening visit.

### Dietary Intervention

The hypothesized optimal diet for MIR is a moderate-fat diet high in MUFA (HMUFA) with a targeted macronutrient composition of 38% of energy from fat (20% MUFA, 8% PUFA, 8% SFA), 48% of energy from carbohydrates (CHO) (30% polysaccharides; 3 g/MJ fiber), and 14% of energy from protein (Table 1). The hypothesized optimal diet for LIR is low in fat, and high in protein (LFHP) and fiber. Energy from CHO is similar between diets. The targeted macronutrient composition of the LFHP diet is composed of 28% of energy from fat (10% MUFA, 8% PUFA, 8% SFA), 48% of energy from CHO (30% polysaccharides; >4 g/MJ fiber), and 24% of energy from protein (Table 1).

**Table 1:** Targeted nutrient composition of the HMUFA and LFHP diet.

	**HMUFA**	**LFHP**
**Fat (en%)**	38	28
Monounsaturated fat	20	10
Polyunsaturated fat	8	8
Saturated fat	8	8
**Protein (en%)**	14	24
Animal-based, % of total protein	45	60
Plant-based, % of total protein	55	40
**Carbohydrates (en%)**	42	42
Mono- and disaccharides	12	12
Polysaccharides	30	30
Fiber, g/MJ	3	>4
Alcohol	<3	<3

*En%, energy percentage of total energy intake; MJ, megajoule*.

The dietary intervention strategy is based on intensive dietary counseling and provision of key products. Before the start of the intervention, a short dietary history is performed to assess the participants' dietary habits and preferences. This information is used to individualize the dietary plan and counseling accordingly. Participants are assigned to one of eight energy groups ranging from 6 to 13 MJ/d according to their estimated individual energy requirement, which is calculated by averaging self-reported energy intake from the FFQ with the product of the predicted BMR, as calculated with Schofield equations (35), and self-reported physical activity level.

At the start of the intervention period, participants receive verbal and written instructions on their dietary plan, which lists both types and quantities of foods that they are required to consume daily or weekly in order to meet the targeted nutrient composition of the assigned diet. The instructions include guidance on what types of foods to choose and avoid within all food groups (e.g., what grain products are [not] allowed; what type and cut of meat or poultry is [not] allowed). Intake of so-called free-food items (e.g., from caloric sweeteners, sweets, sweet spreads, cookies, fruit juice, sugar-sweetened and/or alcoholic beverages) is restricted to 5–10% of energy intake in both diets. The individual dietary plans include a number of “points” per day that have to be “spent” on such foods.

Key products that largely distinguish the two diets with regards to macronutrient composition are provided in pre-measured amounts. For the HMUFA diet, key products include olive oil, olives, olive tapenade, and low-fat margarine with olive oil. Key products for the LFHP diet include low-fat yogurt and quark, reduced-fat cheese, very low-fat spread, pumpkin seeds, baking margarine with olive oil, and a dietary fiber supplement (2 g β-glucan per 6 g, PromOat®, DSM Nutritional Products, Basel, Switzerland) providing 6–12 g of additional fiber per day. Participants are instructed to finish a certain amount of every provided product each day. Apart from the fiber supplement, all products are commercially available. Alcohol consumption is restricted to ≤ 1 glass/day, in agreement with the current Dutch dietary guidelines (36).

Throughout the intervention period, participants visit the research facilities every week for a 15- to 30-min individual dietary counseling session with a dietitian or research nutritionist to monitor diet adherence, body weight, and adverse events using a semi-structured interview. These sessions are supported by advice via e-mail or telephone if needed. To be able to assess the effects of the dietary intervention on metabolic health parameters, independent of changes in body weight, we aim to keep participants on a stable body weight throughout the study. In case of weight loss or gain, participants are reassigned to a higher or lower energy group to prevent further weight change. To promote overall diet adherence, participants are allowed to deviate from their dietary plan on three individual days throughout week 2–10 of the intervention period. Participants are asked to keep a food record (FR) on these days.

During the COVID-19 restrictions, the weekly on-site visits are replaced by telephone or video-call consultations, key products are home-delivered by courier, and participants weigh themselves at home.

Dietary compliance is assessed by three unannounced 1-day FR with the mobile app “Traqq” (37) on 2 non-consecutive weekdays and 1 weekend day. Participants are provided with written and face-to-face instructions on how to record dietary intake. Participants that do not have a smartphone complete the FRs on paper, which are later entered into the app by the researcher.

### Measurements

**Figure 2:**
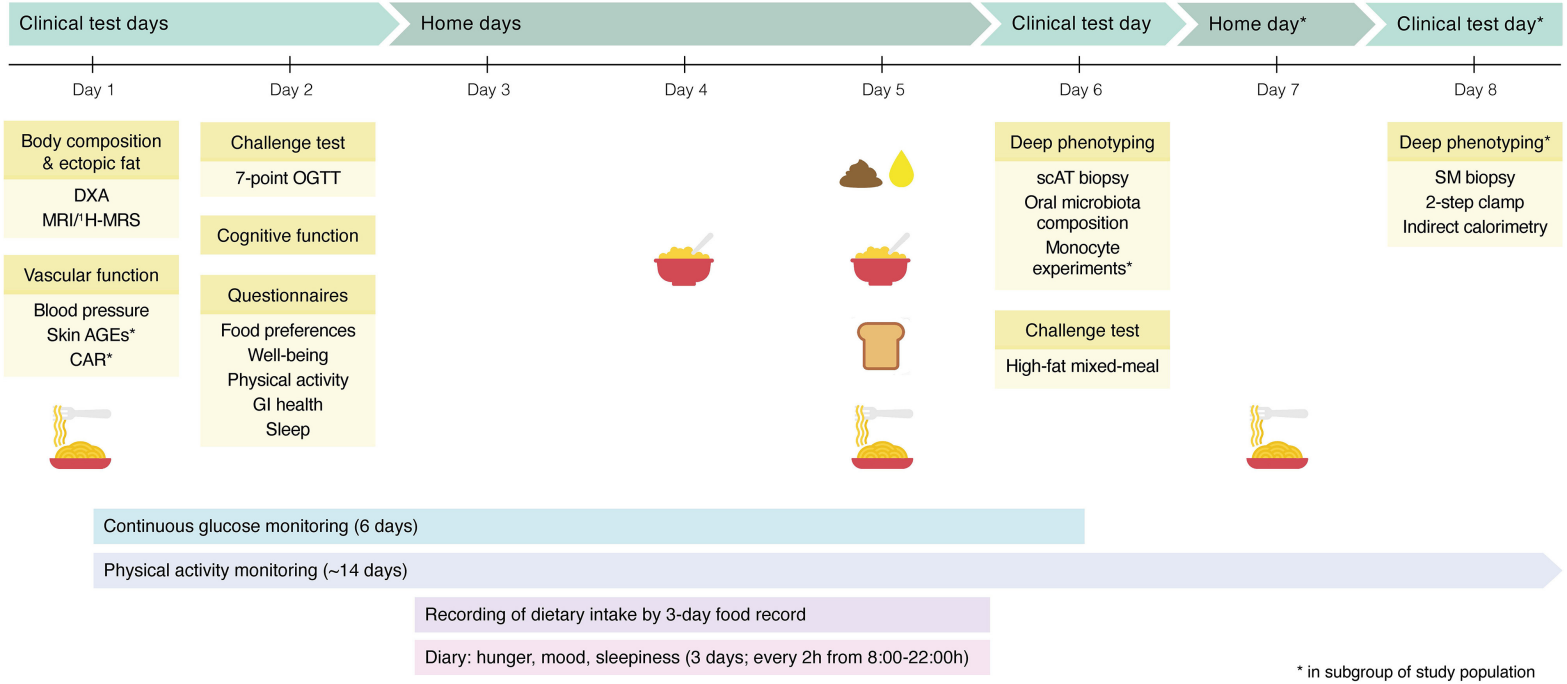
Graphical overview of the pre- and post-intervention characterization week. The characterization week contains multiple clinical test days, during which participants are extensively phenotyped. In addition, blood glucose and physical activity are continuously monitored. At home, participants record their dietary intake and feelings of well-being, collect a fecal sample and 24-h urine, and consume a standardized breakfast on day 4, and on day 5, participants have a full day of standardized meals and snacks, including the standardized breakfast. In a subgroup of the study population, additional measurements are performed. DXA, dual-energy X-ray absorptiometry; MRI, magnetic resonance imaging; ^1^H-MRS, proton magnetic resonance spectroscopy; AGEs, advanced glycation endproducts; CAR, carotid artery reactivity; OGTT, oral glucose tolerance test; GI, gastrointestinal; scAT, subcutaneous adipose tissue; SM, skeletal muscle.

**Figure 3:**
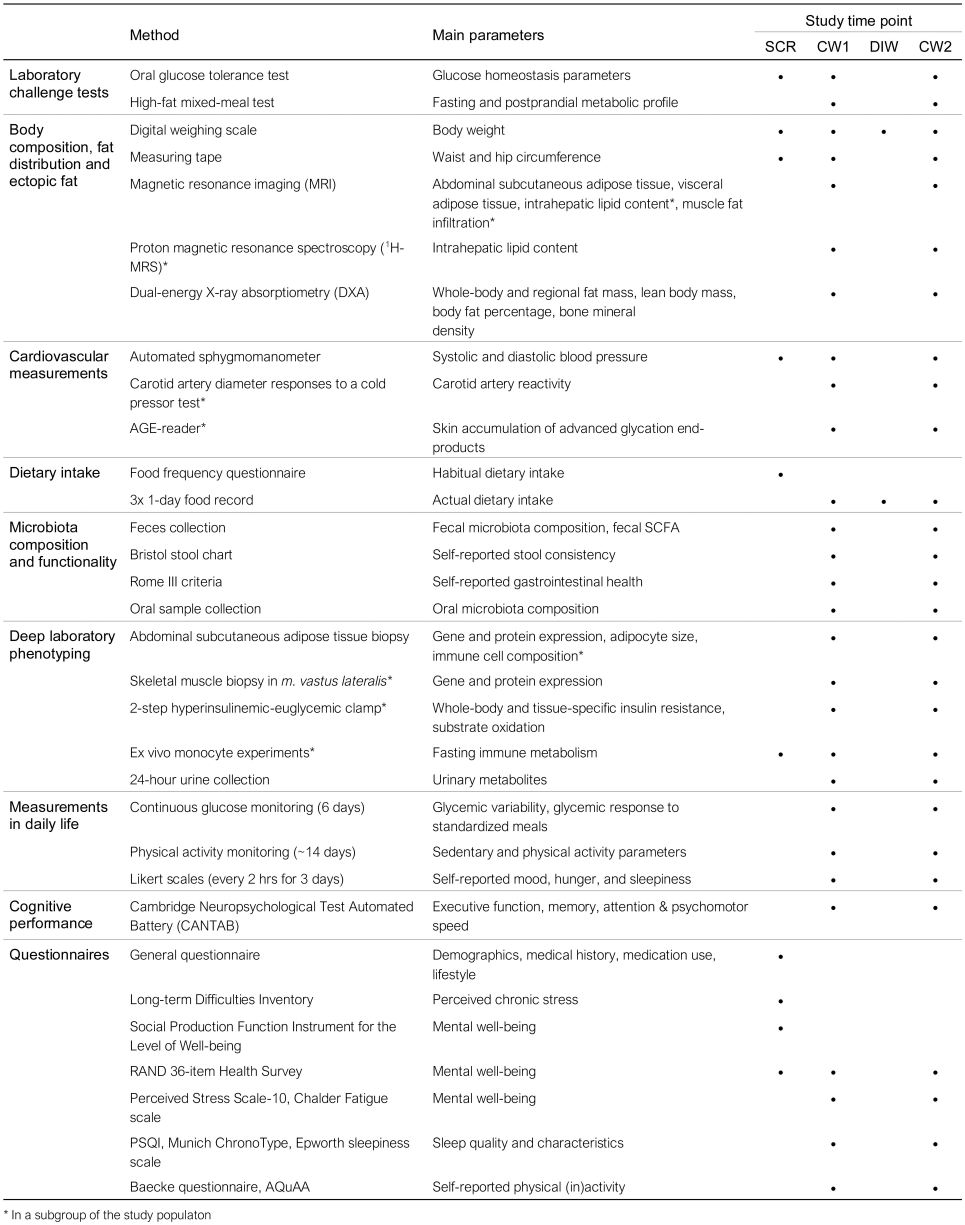
Overview of all measurements performed within the PERSON study. *Performed in a subgroup of the study population. SCR, screening visit; CW, characterization week; DIW, dietary intervention week.

In the week before start of the intervention and in the last week of the 12-week intervention, participants are extensively phenotyped during a “characterization week” (Figure 2). This week includes three or four (depending on study center and participation in additional subgroup measurements) clinical test days and three at-home days. Participants wear a continuous glucose monitor (CGM) and activity monitor throughout the characterization week. During the clinical test days, participants undergo extensive laboratory testing, which includes challenge tests, body composition analysis, vascular measurements, tissue biopsies, a cognitive test, and questionnaires. During the at-home days, participants record dietary intake and feelings of well-being, consume various standardized meals, and collect feces and urine. An overview of all measurements can be found in Figures 2, 3 and are described in more detail below.

On the clinical test days, participants are instructed to travel to the facility by car or public transport. The day prior to and during the characterization weeks, participants are requested to refrain from alcohol and vigorous physical activity. In the week before the baseline characterization week, participants record their dietary intake for three random days (2 week days and 1 weekend day) using the mobile app “Traqq” (37).

#### Laboratory Challenge Tests

**Figure 4:**
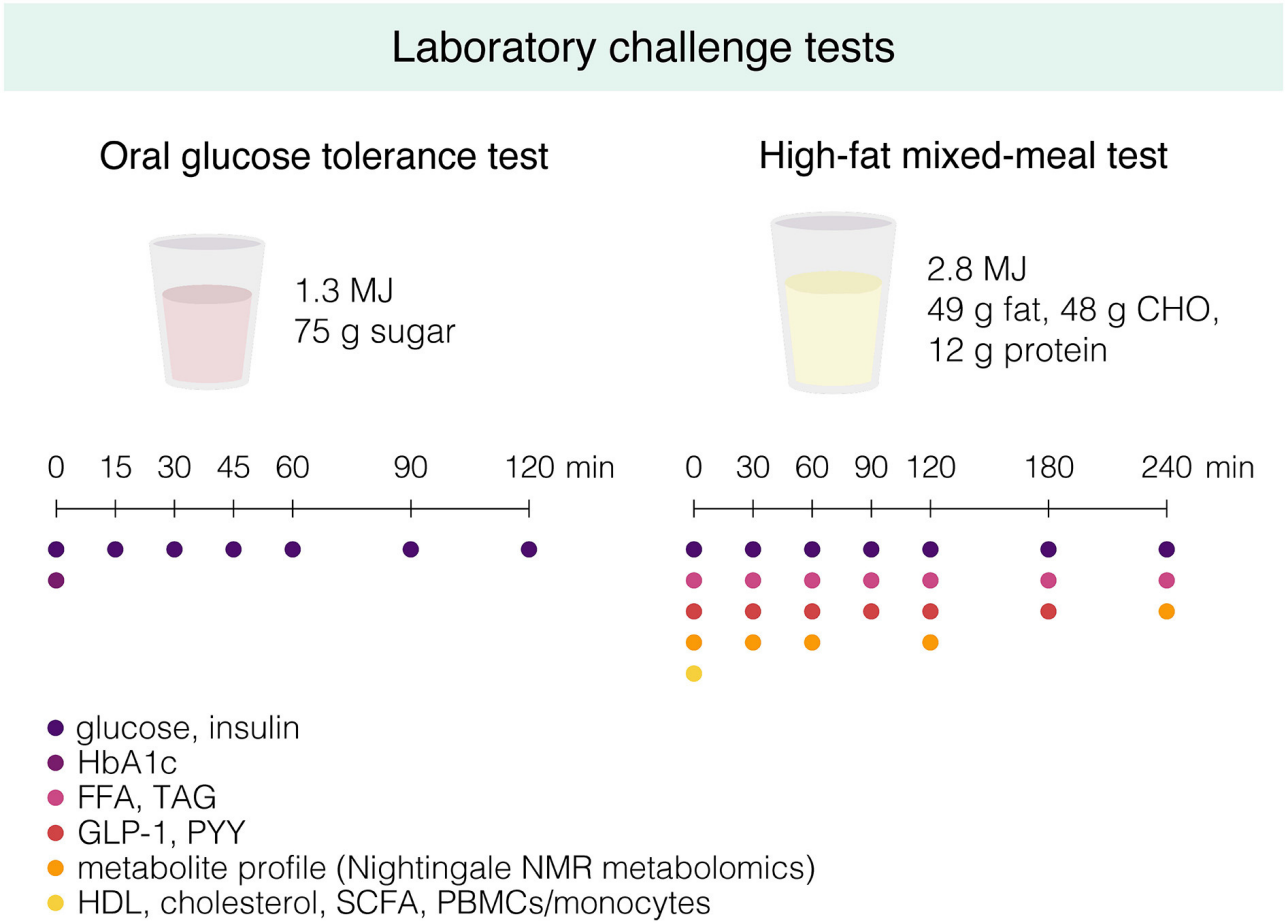
Graphical overview of the oral glucose tolerance test and the high-fat mixed-meal (HFMM) test that are performed during the pre- and post-intervention characterization week. Participants are instructed to drink the glucose drink or HFMM within 5 min, and fasting and postprandial blood samples are drawn at the indicated timepoints for determination of the indicated metabolites. CHO, carbohydrates; HbA1c, hemoglobin A1c; FFA, free fatty acids; TAG, triglycerides; GLP-1, glucagon-like peptide 1; PYY, peptide YY; NMR, nuclear magnetic resonance; HDL, high-density lipoprotein; SCFA, short-chain fatty acids; PBMCs, peripheral blood mononuclear cells.

A 7-point OGTT is performed according to the same procedures used at screening (see “Screening”) (Figures 3, 4). Participants consume a standardized low-fat macaroni meal (30% of energy intake [en%] fat, 49 en% CHO, 21 en% protein; 1,560–2,460 kJ, depending on energy group) the evening before the OGTT, after which they remain fasted until the OGTT. The macaroni meal is prepared in the university kitchen. A fasting blood sample is drawn for determination of glycated hemoglobin (HbA1c) by the hospital laboratories of MUMC+ and Ziekenhuis Gelderse Vallei, Ede, the Netherlands.

On a separate clinical test day, at least 4 days after the OGTT, a high-fat mixed-meal (HFMM) challenge test is performed after a 12-h overnight fast (Figures 3, 4). Participants again consume the standardized low-fat macaroni meal the evening before the test. The liquid HFMM (350 g containing 2.8 MJ, 49 g [64 en%] fat, 48 g [29 en%] carbohydrate, 12 g [7 en%] protein) is prepared in the university kitchen using whipped cream ice cream, whipped cream, full-fat milk, and sugar (Supplementary Table 2). An intravenous cannula is inserted in the antecubital vein for blood sampling. At least 30 min following insertion of the catheter, a fasting blood sample is drawn (*t* = 0 min). Subsequently, participants are asked to consume the liquid HFMM within 5 min and postprandial blood samples are drawn at *t* = 30, 60, 90, 120, 180, and 240 min for determination of glucose, insulin, free fatty acids (FFA), triacylglycerol (TAG), glucagon-like peptide 1 (GLP-1), and peptide YY (PYY) (Figure 4). Total cholesterol and HDL cholesterol are determined in fasting serum. Extensive plasma metabolite profiling is performed in samples from *T* = 0, 30, 60, 120, and 240 min by high-throughput nuclear magnetic resonance (NMR) metabolomics (Nightingale Health Ltd., Helsinki, Finland) (38). Buffy coat is collected from fasting blood for later DNA isolation and genotyping. At each blood drawing, participants rate their hunger, fullness, satiety, thirst, and desire to eat on a 100-mm Visual Analog Scale (VAS), anchored at the extremes “not at all” to “extremely.”

#### Cardiovascular Markers

Blood pressure is assessed according to the same procedures used at screening. In a subgroup of participants, vascular function is assessed by measuring carotid artery reactivity (CAR) to a cold pressor test (CPT) (39). After 10 min of rest in supine position, the participant's left hand is submerged in a bucket of icy water ( ≤ 4°C) for 3 min. The diameter of the left common carotid artery is monitored during a 1-min baseline assessment and continuously during the 3-min CPT using ultrasound (Terason uSmart 3300, Burlington, MA, USA). Wall-tracking and edge-detecting software is used to calculate the diameter after completion of the test. To confirm sympathetic stimulation, blood pressure is measured after the supine rest, 1-min and 2- min after the start of the CPT, and directly after completion of the CPT (Omron M6 Comfort, Omron healthcare Co., Ltd., Kyoto, Japan).

In a subgroup, skin accumulation of advanced glycation end-products (AGE) is measured by skin autofluorescence (AF) using the automated AGE reader (DiagnOptics Technologies B.V., Groningen, the Netherlands). Skin AF is measured at three slightly different places on the volar side of the dominant arm, avoiding impurities of the skin such as scars and birthmarks. Participants are instructed to not apply any creams, lotions, or sunscreen on their arms on the day of the measurement.

#### Body Composition, Fat Distribution, and Ectopic Fat Deposition

Body weight is measured in underwear, and waist and hip circumference are measured according to the procedures described earlier (see “Screening”). Whole-body and regional fat mass, fat percentage, lean body mass, and bone mineral density are assessed using dual-energy X-ray absorptiometry (DXA), while participants are fasted for ≥2 h (MUMC+, Discovery A, Hologic; WUR, Lunar Prodigy, GE Healthcare) (Figure 3).

At MUMC+, a whole-body scan is made after a ≥2 h fast with a 3T magnetic resonance imaging (MRI) scanner (3T MAGNETOM Prisma fit, Siemens Healthcare), using a radiofrequency transmit/receive body coil at Scannexus, Maastricht, the Netherlands. Analyses are performed using a computational modeling method [AMRA Medical AB, Linköping, Sweden (40)] for quantification of abdominal subcutaneous adipose tissue (ASAT), visceral adipose tissue (VAT), thigh muscle volume, intrahepatic lipid content (IHL), and muscle fat infiltration (MFI) in the anterior thighs (Figure 3).

At WUR, IHL and abdominal fat distribution are assessed with proton magnetic resonance spectroscopy (^1^H-MRS) and MRI, respectively, on a 3T whole-body scanner (Siemens, Munich, Germany; Philips Healthcare, Best, the Netherlands from November 2020 onwards). MRI measurements are performed after a ≥2 h fast at hospital Gelderse Vallei, Ede, the Netherlands. Spectra for determination of IHL are obtained from a 30 × 30 × 20 mm voxel placed in the right lobe of the liver, avoiding blood vessels and bile ducts. Participants are instructed to hold their breath when spectra are acquired to reduce respiratory motion artifacts. Spectra are post-processed and analyzed using the AMARES algorithm in jMRUI software. Abdominal fat distribution is evaluated as subcutaneous (ASAT) and visceral adipose tissue (VAT) areas in the abdomen, which are quantified in singles-slice axial T1-weighted spin echo transverse images at the inter-vertebral space L3-L4 using the semi-automatic software program HippoFatTM (41).

#### Microbiota Composition and Functionality

During one of the at-home days in the characterization week, participants collect fecal samples (Figures 2, 3). The samples are stored in the participants' home freezer for maximal 72 h before the visit to the research facilities. Participants rate stool consistency of the sample using the Bristol stool scale (42). Fecal microbiota composition is determined by 16S rRNA sequencing as described elsewhere (43).

During the HFMM challenge test, fasting and postprandial blood samples are collected for determination of plasma concentrations of GLP-1 and PYY (Figure 3). Fecal concentrations and fasting plasma levels of gut microbiota-derived short-chain fatty acids (SCFA) acetate, propionate and butyrate are determined using optimized LC-MS protocols (44).

Data on self-reported gastrointestinal health are collected by a questionnaire based on the Rome III criteria (45). The questionnaire includes questions on presence of gastrointestinal complaints (i.e., abdominal pain, obstipation, bloating), defecation frequency, and stool consistency (Figure 3).

In addition, oral samples are collected for microbiological and metabolite analyses. Participants are asked to rinse the oral cavity thoroughly for 30 s with 10 ml of sterile 0.9% saline and expectorate the rinse in a tube. The tube is kept on ice, vortexed and the rinse is aliquoted, snap-frozen in liquid nitrogen and stored at −80 °C for later analysis. Participants are instructed to refrain from oral hygiene in the morning of the sampling day. The composition of the oral microbiome is determined by 16S rRNA sequencing (46).

#### Deep Laboratory Phenotyping

##### Abdominal Subcutaneous Adipose Tissue Biopsy

On the morning of the HFMM, an abdominal SAT biopsy is collected 6–10 cm lateral from the umbilicus under local anesthesia (1% lidocaine) by needle biopsy. The samples are washed with saline to remove blood clots. A portion of tissue is fixed overnight at 4°C in 4% paraformaldehyde and embedded in paraffin for histological sections to determine adipocyte morphology. In a subgroup of participants, at baseline only, ~0.7 g of fresh AT is used for fluorescence activated cell sorting (FACS) analysis. In short, the stromal vascular fraction is isolated from the AT and stained with a cocktail of antibodies for flow cytometry for identification of immune cells (47). The remaining tissue is snap-frozen in liquid nitrogen and stored at −80 °C for later analyses of targeted gene and protein expression.

##### Skeletal Muscle Biopsy

In a random subgroup of participants at MUMC+ (*n* = 60 in total; *n* = 15 per intervention group), a skeletal muscle (SM) biopsy is collected and a two-step hyperinsulinemic-euglycemic clamp is performed on a separate clinical test day at the end of the characterization week (Figure 3). The skeletal muscle biopsy is taken from the *m. vastus lateralis* under local anesthesia using the Bergström biopsy needle method (48). After removal of blood and fat tissue, a portion of the biopsy is snap-frozen in melting isopentane and stored at −80 °C for biochemical analyses. The remaining tissue is snap-frozen in liquid nitrogen and stored at −80 °C for later gene and protein expression analyses.

##### Two-Step Hyperinsulinemic-Euglycemic Clamp

After the SM biopsy, whole-body and tissue-specific insulin sensitivity are assessed by the gold standard two-step hyperinsulinemic-euglycemic clamp (49). At *t* = −120 min, primed D-[6.6-^2^H_2_] glucose tracer is started and infused continuously at 0.04 mg/kg/min, to allow calculations of rates of endogenous glucose production (EGP), glucose appearance (Ra), and glucose disposal at basal conditions. At *t* = 0, a low primed constant co-infusion of insulin at 10 mU/m^2^/min is started for 3 h for determination of hepatic insulin sensitivity. At *t* = 180 min, the primed constant infusion of insulin is increased to 40 mU/m^2^/min for 2.5 h to inhibit EGP and measure muscle insulin sensitivity. Arterialized blood is frequently sampled from the superficial dorsal hand vein during the insulin infusion to measure glucose concentrations, which are maintained at ~5.0 mmol/L by a co-infusion of 20% glucose at variable rate (GIR). Substrate utilization is measured for 30 min during the basal, low insulin, and high insulin infusion using indirect calorimetry by ventilated hood (Omnical, Maastricht Instruments, Maastricht). Resting metabolic rate (RMR), fat and carbohydrate oxidation are calculated according to the equations of Weir and Frayn (50, 51). The clamp is performed after an overnight (≥12 h) fast and participants consume the standardized macaroni meal the evening before the clamp.

##### Fasting Immune Metabolism

At WUR only, circulating peripheral blood mononuclear cells (PBMCs) are isolated from fasted blood samples collected at the HFMM test (Figure 3). In addition, in a random subgroup (*n* ~ 200), PBMCs are also isolated from fasted blood samples collected at screening. PBMCs are isolated by density gradient isolation using CPT tubes (BD vacutainer, cat. no. 362753). Monocytes are subsequently obtained by MACS (magnetic activated cell sorting) positive selection using CD14 MicroBeads (Miltenyi Biotec, cat no. 130-050-201). Part of the monocytes are exposed overnight (24 h) to the inflammatory stimuli lipopolysaccharide (LPS) (10 ng/mL, sigma, cat. no L6529) and P3C (10 ug/mL, EMC collections, cat. no. L2000). Functional properties of monocytes are determined after treatment by measuring the release of cytokines including IL-6, IL-1b and CXCL8 (R&D DuoSet ELISA, cat. no. DY206; DY201; DY208). The metabolic potential of monocytes is measured in real-time experiments (inflammatory cell activation test and glycolytic stress test) using the Seahorse apparatus (Agilent Technologies) in screening samples only.

##### 24-h Urine Collection

Participants collect 24-h urine in 2-3 liter containers containing 5 ml/L of 4 mM hydrochloric acid (HCl). Urine collection starts after the first voiding on the morning of the home-day with only standardized meals and finishes 24 h later on the morning of the HFMM. Participants are asked to store the containers in a cool place, preferably a refrigerator, and bring the containers to the facilities on the day of the HFMM. The urine of each participant is mixed, weighted, aliquoted, and stored at −80 °C for later analysis.

#### Measurements in Daily Life and At-Home Days

##### Continuous Glucose Monitoring

At the start of the characterization week, a CGM (Medtronic iPro2 with Enlite sensor) is placed lateral to the umbilicus for 6 days of continuous interstitial fluid glucose measurements (Figure 2). The CGM data are calibrated according to the manufacturer's instructions with four daily capillary glucose self-measurements using a blood glucose meter (Contour XT, Ascensia Diabetes Care).

To assess glycemic variability and glycemic responses to standardized meals, on one of the home-days, participants consume a standardized breakfast, and on another home-day, participants have a full day of standardized meals and snacks, including the standardized breakfast (Figure 2; Supplementary Tables 3, 4). Participants are instructed to consume the meals according to standardized instructions including time frames, to fast for 2 h after the breakfast, and to only drink water alongside the standardized meals.

##### Physical Activity Assessment

Physical activity is continuously monitored for ~14 days during both the characterization weeks and ~7 days in free-living conditions–either starting with the characterization week at baseline, or ending with the characterization week in week 12 (Figure 2)–using a triaxial accelerometer (activPAL3™ micro, PAL Technologies Ltd., Glasgow, Scotland, UK) attached to the middle of the right thigh. Participants keep a diary to record the times they go to sleep and wake up while wearing the monitor. Sedentary and physical activity parameters are quantified with a modified version of the script of Winkler et al. (52), using the sleeping and waking times as input.

##### Dietary Intake, Hunger, Mood, and Sleepiness

During the 3 at-home days, participants record their dietary intake including consumption of the standardized meals using the mobile app “Traqq” (37). In addition, participants are asked to report on hunger, mood, and sleepiness every 2 h from 8:00 to 22:00 h (Figure 2). Hunger is rated on an 11-point Likert scale ranging from “not hungry” to “very hungry.” Self-reported mood is assessed with an adapted form of the Multidimensional Mood Questionnaire (MDMQ) (53). The 7-point scale consists of six bipolar items to assess the three basic dimensions of mood valence, calmness, and energetic arousal: tired/awake, satisfied/dissatisfied, agitated/calm, full of energy/without energy, unwell/well, and relaxed/tense. Sleepiness is rated on the 9-point Karolinska Sleepiness Scale, with labels ranging from “extremely alert” to “very sleepy, great effort keeping awake, fighting sleep” (54, 55).

#### Cognitive Performance

Cognitive performance is assessed in the domains of executive function, memory, and attention & psychomotor speed using the Cambridge Neuropsychological Test Automated Battery (CANTAB) (56). Executive function is evaluated with the multitasking test and spatial span test; memory with the delayed matching to sample test and paired associates learning test; and attention and psychomotor speed is assessed with the motor screening task and reaction time task. Each test is preceded by standardized instructions and a practice round for familiarization. Participants consume a standardized brunch containing of bread with cheese and/or ham and a caffeine-free drink before performing the test battery.

#### Self-Reported Food Preferences, Eating Rate, Sleep, Well-Being, and Physical (In)-activity

After the CANTAB, participants complete the computer-based Macronutrient and Taste Preference Ranking Task (MTPRT) for assessment of food preferences (57). The task assesses liking and ranking for 32 food products that are categorized as high in carbohydrates, high in fat, high in protein, or low-calorie, as well as either sweet or savory. Furthermore, participants rate their eating rate in comparison to others on a 5-point Likert scale with labels ranging from “very slow” to “very fast” (Figure 3).

In addition, during one of the clinical test days, participants provide information on general well-being, sleep characteristics, and physical (in)activity by questionnaire (Figure 3). Mental well-being is assessed using the RAND-36 (30) and perceived stress is measured with the 10-item Perceived Stress Scale (PSS-10) (58). Physical and mental fatigue are assessed using the 14-item Chalder fatigue scale (59). Sleep quality is assessed with the 10-item Pittsburgh Sleep Quality Index (60) and sleep duration and chronotype are derived from the Munich ChronoType Questionnaire (61). Daytime sleepiness is assessed with the 8-item Epworth Sleepiness scale (62) (Figure 3).

Self-reported habitual physical activity and sedentary behavior are assessed using the Baecke questionnaire (63) and the Activity Questionnaire for Adults and Adolescents (AQuAA) subscale “sedentary leisure time activities” (64), respectively. In addition, physical activity self-efficacy is measured with 5 items from a health specific self-efficacy scale (65) and physical inactivity temptations are assessed using the 5-item subscale “competing demands” from the Temptation to not Exercise Scale (66), extended with the item “How tempted are you not to exercise and be sedentary while being on a business trip?”.

#### Biochemical Analyses of Blood Samples and Biobanking

A wide range of biological samples are collected in the present study, including blood plasma and serum, SAT, SM tissue, feces, urine, saliva, and PBMCs. EDTA (Becton Dickinson, Eysins, Switzerland) tubes are centrifuged at 1,200 g, 4°C for 10 min and plasma is aliquoted subsequently. Serum tubes are left at room temperature for at least 30 min to allow clotting after sampling and centrifuged at 1,200 g, 20°C for 10 min before aliquoting of serum. All biological samples are snap-frozen in liquid nitrogen and stored at −80°C until analysis. Samples from both centers are analyzed at central laboratories. Plasma glucose, insulin, and FFA are measured on a Cobas Pentra C400 using ABX Pentra Glucose HK CP reagens (Horiba ABX Diagnostics, Montpellier, France), ELISA (Meso Scale Discovery, Gaithersburg, USA), and NEFA HR (2) reagens (2) (Wako chemicals, Neuss, Germany), respectively. Serum TAG, total cholesterol, and HDL cholesterol are measured on a Cobas Pentra C400 using ABX Pentra Triglycerides HK CP reagens, ABX Pentra Cholesterol CP reagens, and ABX Pentra HDL Direct, respectively. During the HFMM challenge test, fasting and postprandial blood samples are collected in EDTA tubes and aprotinin tubes containing dipeptidyl peptidase-IV inhibitor (Milipore Merck, Billerica, MA, USA) for determination of plasma GLP-1 and PYY, respectively. Total GLP-1 immunoreactivity is assessed using an antiserum that reacts equally with intact GLP-1 and the primary (N-terminally truncated) metabolite as previously described (67). PYY concentrations are determined with a commercially available radioimmunoassay for Human PYY (3-36) (Millipore Corporation, MA, USA).

### Data Management

Data are collected on paper case report forms (CRF) and are entered in an electronic CRF designed for the study, using the web-based data capturing platform Caster EDC (68) that is compliant with good clinical practice (GCP) requirements. All relevant raw and processed data (e.g., from blood analyses, DXA scan) are also added to the eCRF in Castor EDC. Data entered in the eCRF are checked against the paper CRF by a study team member that did not enter the data. Data are collected and stored according to the FAIR (Findability, Accessibility, Interoperability, and Reusability) principles (69). A central data manager monitors data entry of both centers, performs data cleaning, and ensures that inaccurate or missing data are addressed.

### Sample Size Calculation

Based on previous data, we expect a greater improvement in disposition index in participants receiving their hypothesized optimal diet compared to those receiving their hypothesized suboptimal diet (10). Data from the previously published DiOGenes study (18) as well as the CORDIOPREV-DIAB Study (18) were used to calculate an average standardized effect size from the difference in outcome values between the optimal and suboptimal diets in those studies. For DiOGenes, the low vs. high GI diets during the weight regain period were used and for CORDIOPREV the Mediterranean vs. low fat-high complex carbohydrate diets were used, in interaction with either MIR or LIR. With a power of 90%, two-sided alpha of 5% and a standardized effect size of 0.46, a total sample size of 202 was calculated using the statistical analysis software R. Taking into account a drop-out rate of 15%, 240 subjects will be included.

### Statistical Analyses

In this paper, preliminary screening data from May 2018 to March 2020 are included. Baseline characteristics were compared between the four IR phenotypes (No MIR/LIR, MIR, LIR, combined MIR/LIR), using one-way ANOVA with Bonferroni *post-hoc* pairwise comparisons for numerical data (mean ± SD), and using Fisher's exact test for categorical data (%). Parameters of glucose homeostasis from the OGTT and dietary intake data from the FFQ were log-transformed due to non-normality, and differences between the IR phenotypes were tested using ANCOVA with adjustment for sex and Bonferroni *post-hoc* pairwise comparisons. Statistical analyses were performed in SPSS (version 25.0). Differences in glucose and insulin responses following the OGTT between the IR phenotypes were tested using linear mixed-effects models (LMM) with Bonferroni *post-hoc* pairwise comparisons. The time courses of glucose and insulin were modeled with third-order (cubic) orthogonal polynomials. The effect of IR phenotype on all time terms and sex were included as fixed effects with participant random effects on all time terms. The adequacy of the higher order polynomials was assessed with a likelihood-ratio test between nested models. The covariance matrix of the residuals was modeled as an unstructured matrix and model parameters were estimated using maximum likelihood estimation in all models. Estimated marginal means (EMM) with the degrees of freedom and corresponding *p*-values were estimated using Satterthwaite's method. All mixed-effects models were implemented using the “lmer” function of the lme4 package and EMMs were computed using the emmeans package in R (version 3.3.3, The R foundation for Statistical Computing, http://www.r-project.org/).

## Results

**Figure 5:**
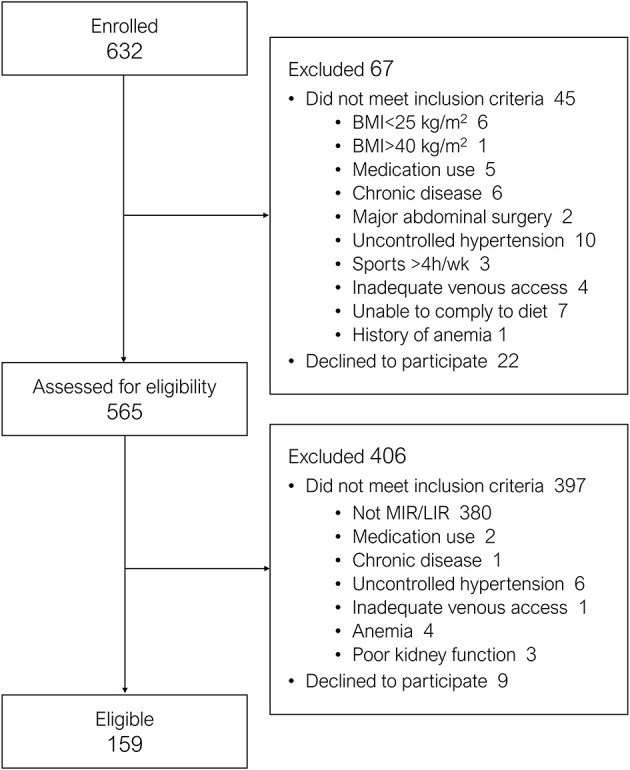
Flowchart of participant enrollment and eligibility from March 2018 to March 2020.

Between May 2018 and March 2020, 632 individuals were enrolled, of whom 565 were fully screened for eligibility (Figure 5). In total, 40.2% of fully screened individuals were classified as No MIR/LIR, 21.4% as MIR, 10.8% as LIR, and 27.6% as combined MIR/LIR. Here, we present the characteristics of the study participants that have thus far been screened in the present ongoing clinical trial.

### Baseline Characteristics

Baseline characteristics of all participants that completed screening are reported according to IR phenotype in Table 2. Mean age of the four groups (60 – 62 years) was comparable. The proportion of women in the total study population was 59% and was higher in the MIR group (69%) compared to the other groups, but only statistically significantly different from the combined MIR/LIR group. Individuals with combined MIR/LIR had higher BMI, waist circumference, waist-to-hip ratio, systolic and diastolic blood pressure, ALT levels, and use of antihypertensive medication compared to the No MIR/LIR, MIR, and LIR groups. Anthropometric and clinical characteristics were similar between the MIR and LIR group. IFG was most prevalent in the LIR group (11.5%), while IGT was most prevalent in the MIR group (18.2%) and the combined MIR/LIR group (16.7%). The prevalence of newly diagnosed T2DM was 6.6%, 8.3%, 0.0%, and 10.9% in the No MIR/LIR, MIR, LIR, and combined MIR/LIR group, respectively.

**Table 2:** Characteristics of screened participants according to insulin resistance phenotype.

	**No MIR/LIR**	**MIR**	**LIR**	**Combined MIR/LIR**	***P*-value**
	**(*n* = 227)**	**(*n* = 121)**	**(*n* = 61)**	**(*n* = 156)**	
Age (years)	61 ± 9	60 ± 9	61 ± 8	62 ± 8	0.627
Women (%)	59.9	69.4	54.1	52.6^†^	0.031
Weight (kg)	86.6 ± 13.1	86.0 ± 11.2	86.4 ± 11.8	94.0 ± 14.7^§^^†^^‡^	<0.001
BMI (kg/m^2^)	29.2 ± 3.3	29.8 ± 3.1	29.6 ± 3.2	32.2 ± 4.1^§^^†^^‡^	<0.001
Waist circumference (cm)	98.5 ± 10.6	100.4 ± 9.5	99.9 ± 9.5	106.3 ± 11.0^§^^†^^‡^	<0.001
Waist-to-hip ratio	0.91 ± 0.09	0.92 ± 0.08	0.94 ± 0.09	0.95 ± 0.09^§^^†^^‡^	<0.001
SBP (mmHg)	132 ± 17	132 ± 13	132 ± 16	137 ± 16^§^	0.015
DBP (mmHg)	80 ± 11	80 ± 10	80 ± 11	85 ± 10^§^^†^^‡^	<0.001
Hemoglobin (mmol/L)	8.8 ± 0.7	8.7 ± 0.7	8.8 ± 0.7	9.0 ± 0.8^§^^†^^‡^	0.003
Creatinine (μmol/L)	75.0 ± 14.1	73.8 ± 14.1	76.0 ± 16.2	78.4 ± 14.7	0.051
ALT (IU/L)	23 ± 10	27 ± 12	25 ± 9	31 ± 14^§^^†^^‡^	<0.001
AST (IU/L)	22 ± 6	22 ± 7	23 ± 6	25 ± 8^§^^†^	0.002
Use of statins (%)	9.7	6.6	11.5	13.5	0.293
Use of antihypertensives (%)	17.2	15.7	14.8	28.2	0.022
Family history of diabetes (%)	24.7	21.5	18.3	25.2	0.685
Glucose status (%)					<0.001
NGT	78.4	71.1	75.4	62.2	
IFG	6.2	0.8	11.5	2.6	
IGT	7.0	18.2	6.6	16.7	
Combined IFG/IGT	1.8	1.7	6.6	7.7	
T2DM	6.6	8.3	0.0	10.9	
Employment status (%)					0.429
Paid job	44.6	49.2	43.3	36.8	
Retired	39.7	37.5	36.7	42.6	
Other	15.6	13.3	20.0	17.0	
Education level (%)					0.257
Low	17.9	11.8	25.0	21.7	
Intermediate	31.8	39.5	28.3	30.9	
High	50.2	48.7	46.7	47.4	

*Differences between tissue-specific IR groups were assessed using one-way ANOVA with Bonferroni post-hoc pairwise comparisons for numerical data (mean ± SD), and using Fisher's exact test for categorial data (%)*.

§ *Significantly different from No MIR/LIR (p < 0.05)*.

† *Significantly different from MIR (p < 0.05)*.

‡ *Significantly different from LIR (p < 0.05)*.

*BMI, body mass index; SBP, systolic blood pressure; DBP, diastolic blood pressure; ALT, alanine transaminase; AST, aspartate aminotransferase; NGT, normal glucose tolerant; IFG, impaired fasting glucose; IGT, impaired glucose tolerance; T2DM, type 2 diabetes mellitus*.

### Glucose Homeostasis

**Figure 6:**
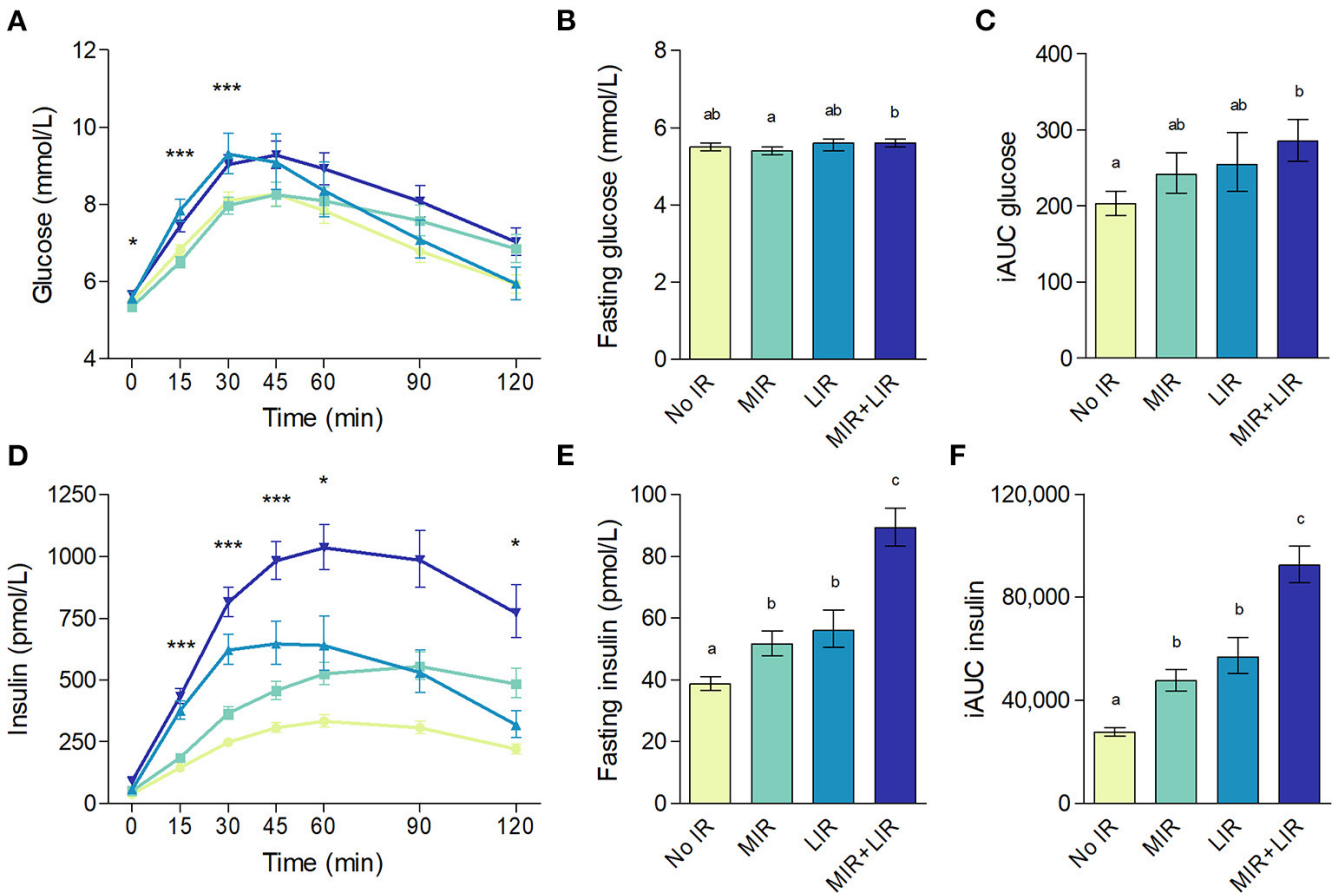
Plasma glucose **(A–C)** and insulin **(D–F)** concentrations during an oral glucose tolerance test according to insulin resistance phenotype. **(A,D)**: data are geometric means with 95% confidence intervals; significant differences for MIR vs. LIR as analyzed using estimated marginal means from linear mixed-effects models with adjustment for sex and Bonferroni *post-hoc* pairwise comparisons are denoted with *(*p* < 0.05) or ***(*p* < 0.001). **(B,C,E,F)**: data are adjusted geometric means with 95% confidence intervals. Different letters (a, b, c, d) indicate significant differences (*p* < 0.05) between IR phenotypes, as tested using ANCOVA with adjustment for sex and Bonferroni *post-hoc* pairwise comparisons.

**Figure 7:**
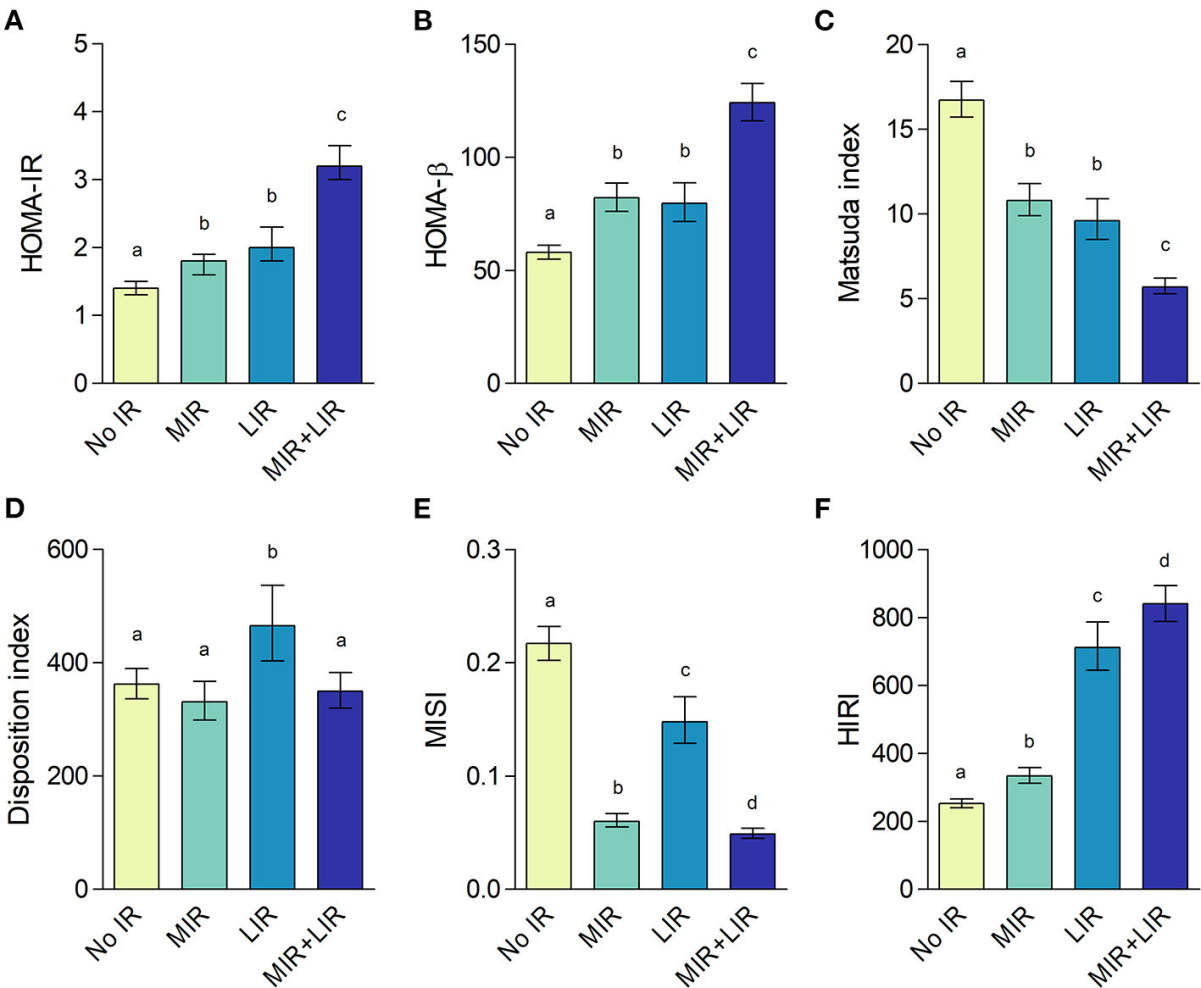
HOMA-IR **(A)**, HOMA-β **(B)**, Matsuda index **(C)**, disposition index **(D)**, muscle insulin sensitivity index **(E)**, and hepatic insulin resistance index **(F)** according to insulin resistance (IR) phenotype. Data are adjusted geometric means with 95% confidence intervals. Different letters (a, b, c, d) indicate significant differences (*p* < 0.05) between IR phenotypes, as tested using ANCOVA with adjustment for sex and Bonferroni *post-hoc* pairwise comparisons.

By definition, both plasma glucose and insulin curves throughout the OGTT differed between the IR groups (*p* < 0.001 for both; Figure 6). Throughout the first 30 min of the OGTT, plasma glucose concentrations were higher in the LIR group compared to the MIR group (Figure 6A). Plasma insulin concentrations were higher in the LIR group compared to the MIR group at timepoints 15 – 60 min, whereas at 120 min, insulin was lower in LIR compared to MIR (Figure 6D). The iAUCs of both glucose and insulin were lowest in the No MIR/LIR group, highest in the combined MIR/LIR group, and comparable between the MIR and LIR group (overall *p* < 0.001; Figures 6C,F), as were HOMA-IR (overall *p* < 0.001; Figure 7A) and HOMA-β (*p* < 0.001; Figure 7B). Similarly, Matsuda index was highest in the No MIR/LIR group, lowest in the combined MIR/LIR group, and comparable between the MIR and LIR group (overall *p* < 0.001; Figure 7C). Disposition index was higher in the LIR group compared to the other groups (overall *p* = 0.002; Figure 7D). Furthermore, by definition, MISI was lowest in the combined MIR/LIR and the MIR group (overall *p* < 0.001; Figure 7E) and HIRI was highest in the combined MIR/LIR and LIR group (overall *p* < 0.001; Figure 7F). All analyses were adjusted for sex. Values of these glucose homeostasis parameters derived from OGTT are reported in Supplementary Table 5.

### Habitual Dietary Intake

FFQ data were available from 549 participants. After exclusion of data from 84 and 4 individuals due to energy under- and overreporting, respectively, data from 461 participants were included in the analyses. The proportion of misreporters did not differ between the IR phenotypes (*p* = 0.411). Energy intake tended to be lower in the MIR group compared to the other groups when adjusted for sex (Table 3; *p* = 0.062). Intake of energy from saturated fat was highest in the combined MIR/LIR group, although only statistically significantly higher compared to the MIR group. Other components of macronutrient composition of habitual dietary intake, expressed as en%, did not differ between the IR phenotypes when adjusted for sex. Alcohol consumption was lower in the combined MIR/LIR group compared to No MIR/LIR (overall *p* = 0.011).

**Table 3:** Habitual dietary intake from FFQ according to insulin resistance phenotype.

	**No MIR/LIR**	**MIR**	**LIR**	**Combined MIR/LIR**	***P*-value**
	**(*n* = 180)**	**(*n* = 98)**	**(*n* = 53)**	**(*n* = 130)**	
Energy (MJ)^a^	9.5 ± 1.0	8.8 ± 1.0	9.5 ± 1.0	9.6 ± 1.0	0.062
Fat (en%)	37.6 ± 0.4	36.8 ± 0.6	37.3 ± 0.8	38.5 ± 0.5	0.127
Monounsaturated fat	13.5 ± 0.2	13.1 ± 0.2	13.3 ± 0.3	13.6 ± 0.2	0.551
Polyunsaturated fat	7.2 ± 0.1	7.1 ± 0.2	7.1 ± 0.2	7.1 ± 0.1	0.946
Saturated fat	13.8 ± 0.2	13.4 ± 0.3	13.8 ± 0.4	14.5 ± 0.3^†^	0.024
Carbohydrates (en%)	41.1 ± 0.5	42.6 ± 0.6	42.1 ± 0.8	40.9 ± 0.5	0.137
Mono- and disaccharides	19.0 ± 0.4	20.0 ± 0.6	19.8 ± 0.8	18.4 ± 0.5	0.150
Polysaccharides	22.1 ± 0.3	22.6 ± 0.4	22.3 ± 0.6	22.5 ± 0.4	0.748
Fiber (g/MJ)	2.6 ± 0.0	2.6 ± 0.1	2.6 ± 0.1	2.5 ± 0.1	0.538
Alcohol (en%)^b^	2.3 ± 0.1	1.8 ± 0.1	2.0 ± 0.1	1.6 ± 0.1^§^	0.011
Protein (en%)	15.7 ± 0.2	15.7 ± 0.2	15.2 ± 0.3	15.8 ± 0.2	0.401
Animal-based, % of total	58.4 ± 0.7	58.4 ± 1.0	57.1 ± 1.3	59.5 ± 0.8	0.475
Plant-based, % of total	41.6 ± 0.7	41.6 ± 1.0	42.9 ± 1.3	40.5 ± 0.8	0.481

*Differences between tissue-specific IR groups were assessed using ANCOVA with adjustment for sex and with Bonferroni post-hoc pairwise comparisons (adjusted mean ± SE)*.

a *Data were logtransformed to improve normality and reported as geometric means*.

b *A constant was added before logtransformation to eliminate zero values*.

§ *Significantly different from No MIR/LIR (p < 0.05)*.

† *Significantly different from MIR (p < 0.05)*.

*FFQ, Food Frequency Questionnaire; MJ, megajoule; en%, energy percentage of total energy intake*.

## Discussion

The purpose of the present article was to describe the study design of the PERSON study and to present preliminary screening results. In the PERSON study, individuals are classified based on IR phenotype at baseline, and randomized to follow a hypothesized optimal or suboptimal diet according to their metabolic phenotype. This study is one of the first randomized double-blind controlled trials in the field of precision nutrition to investigate whether a dietary intervention based on tissue-specific insulin sensitivity improves metabolic health to a greater extent compared to a hypothesized suboptimal diet.

### Dietary Intervention

Both intervention diets prescribed in this study are largely in line with the Dutch dietary guidelines of the Health Council of the Netherlands (36). Data from the FFQ indicated that the habitual dietary intake of our study population did not meet these guidelines. In particular, average fiber intake (2.6 g/MJ) was well below the recommended 3.4 g/MJ, and lower than the targeted fiber intake of 3 g/MJ and 4 g/MJ in the HMUFA and LFHP interventions diets, respectively. In addition, average intake of calories from saturated fat (14 en%) exceeded the <10 en% that is recommended. In our study, prescribed intake of saturated fat and mono- and disaccharides, which is similar between the two interventions diets, is lower than the average habitual intake. Therefore, we expect that on average, participants will benefit from both dietary interventions, regardless of their IR phenotype. Nevertheless, we hypothesize to find greater improvements in glucose homeostasis and related outcomes in study participants that follow the anticipated optimal compared to suboptimal diet.

The hypothesis that dietary macronutrient composition interacts with tissue-specific IR is supported by findings from recent studies. A *post-hoc* analysis of the CORDIOPREV-DIAB study indicated that individuals with predominant MIR had a greater improvement in disposition index on a 2-year Mediterranean diet, while individuals with predominant LIR benefitted more from a diet high in complex carbohydrates and low in fat (18). In addition, individuals with LIR have been shown to have a more detrimental fasting plasma lipid profile (13) and impaired postprandial lipoprotein metabolism following high-fat meals (70) compared to individuals with MIR, which suggests that a low-fat diet may be especially beneficial for individuals with LIR (71). Furthermore, findings from other studies indicate that a high protein diet and high fiber diet may have beneficial effects for individuals with LIR, as both high protein and high fiber diets have been shown to successfully reduce liver fat content (72–75). Liver fat accumulation has been related to decreased suppression of hepatic glucose production in some studies (74, 76), linking liver fat to LIR, although the cause-effect relationship remains to be established. Moreover, increased fiber intake has been shown to improve insulin sensitivity in individuals with IFG but not IGT (77). IFG is characterized mainly by impaired hepatic insulin sensitivity (78, 79), which is in line with observations in our study that individuals with IFG are most often characterized as LIR.

In addition, dietary fat quality may impact skeletal muscle lipid handling. In an acute study, meals high in saturated fat resulted in increased postprandial skeletal muscle fatty TAG extraction and/or reduced intramyocellular lipid turnover compared to meals high in unsaturated FAs in insulin resistant individuals, which was accompanied by a lower postprandial insulin sensitivity (80). Taken together, a “one-size-fits-all” approach with population-wide dietary guidelines may not be optimal for metabolic health for all individuals. A diet targeting tissue-specific IR is expected to increase the effectiveness of dietary interventions with respect to improvements in glucose homeostasis.

Changes in macronutrient composition within the context of an isocaloric diet can improve risk factors for cardiometabolic diseases, independent of weight loss (81). The two diets implemented in the PERSON study differ in macronutrient composition, and are both matched to the participants' individual energy requirements in order to maintain weight stability during the dietary intervention. Throughout the study, participants' body weight is monitored weekly, and adjustments in absolute energy intake, but not diet composition, are made if needed to maintain body weight. We provide key food products, perform unannounced food records, and conduct weekly check-ins with skilled dieticians and researchers, together increasing the incentive to adhere to the diet and the possibility to assess dietary compliance.

### Extensive and Detailed Phenotyping

A strength of the PERSON-study is the extensive and detailed phenotyping of the study participants before and after the dietary intervention. This allows us to comprehensively study the metabolic underpinnings of the metabolic response to the dietary intervention. Next to performing highly standardized metabolic phenotyping in a laboratory setting, we also collect data in free-living conditions. Furthermore, in a subgroup of the study population several additional measurements such as the gold-standard hyperinsulinemic-euglycemic clamp are performed, which allows us to investigate the mechanisms involved in the pathophysiology of tissue-specific IR as well as how these may be affected by the dietary intervention.

Next to detailed metabolic phenotyping, we also collect data on mood, perceived well-being, food preferences and cognitive function. There are indications that blood glucose levels may be an important determinant of mood and cognitive function (19, 21, 82, 83). Additionally, gut microbial profile, which can be modulated by dietary intake, is linked to cognitive function and mood via the gut-brain axis (84, 85). Hence, by improving glucose homeostasis and metabolic health with a dietary intervention, individuals may also experience short-term benefits related to mental and emotional well-being and performance. Such directly perceivable benefits are expected to motivate individuals to better adhere to dietary advice.

In addition, the large amount of collected data will allow for the application of computational techniques to elucidate the inter-individual differences in glucose homeostasis and derive new functional insights. Both mechanistic and data-driven computational modeling approaches have been employed to expand on the physiological properties underlying meal responses (6, 7, 86). The frequently-sampled time series of metabolites (e.g., glucose, insulin) from the OGTT and continuous glucose monitoring will be used to construct models of short-term postprandial dynamics, facilitating the assessment of individuals' capacity to regulate glucose levels in response to a meal. The detailed phenotypic information can be integrated using machine-learning models to derive a comprehensive model of glucose homeostasis. The data generated in the PERSON study will enable such computational methods to progress the field of precision nutrition.

### Preliminary Screening Data

Tissue-specific or whole-body IR (either MIR, LIR or combined) was prevalent in ~60% of the population, which is similar to the reported prevalence of 65% in DMS (16). The prevalence of LIR in this study was lower as compared to DMS (11 vs. 17%, respectively). This can possibly be partly explained by the higher proportion of women in the PERSON study compared to DMS (59 vs. 44%, respectively), since LIR is less prevalent in women than men. Sexual dimorphism in glucose homeostasis and IR is well-recognized and has been linked to differences in relation to hormonal status, lipid handling and inflammatory profile (87), but does require further investigation. These data emphasize that future analyses within the PERSON study should also take sex-specific effects into account.

As expected based on the formulas used to classify MIR and LIR, our preliminary screening data confirmed that both MIR and LIR are related to worse glucose homeostasis compared to individuals without MIR or LIR, in line with observations from DiOGenes and DMS (16, 22). Interestingly, however, the majority of individuals with MIR and LIR (71 – 75%) were classified as normal glucose tolerant. Classical cutoff values only including plasma glucose levels may fail to detect important metabolic impairments related to insulin action, especially in early stages of disease development, while these disturbances are well-known to be highly predictive for the development of cardiometabolic diseases later in life (88, 89). Identification of metabolic impairments at an early stage before the onset of dysglycemia creates an important window of opportunity to use lifestyle interventions such as dietary modulation in order to delay or prevent further glycemic deterioration and progression to cardiometabolic disease.

## Conclusion

The PERSON study is one of the first double-blind, randomized trials in the field of precision nutrition to investigate the effects of a more personalized dietary intervention based on tissue-specific insulin resistance phenotype, on metabolic health outcomes at the functional and molecular level, mental performance and perceived well-being. The high prevalence of tissue-specific IR in adults with overweight and obesity highlights the relevance of investigating the effects of targeted dietary approaches in order to define more optimal diets to improve glucose homeostasis, thereby preventing or delaying the development of cardiometabolic diseases. The PERSON study is expected to contribute knowledge on the effectiveness of targeted nutritional strategies to the emerging field of precision nutrition and enhance the understanding of the complex etiology of generalized and tissue-specific IR.

## Data Availability Statement

The datasets presented in this article are not readily available because the data are part of an ongoing study. Requests to access the datasets should be directed to Ellen Blaak, e.blaak@maastrichtuniversity.nl.

## Ethics Statement

The studies involving human participants were reviewed and approved by the Medical Ethics Committee of MUMC+, Maastricht, The Netherlands. The patients/participants provided their written informed consent to participate in this study.

## Author Contributions

EF, GG, LA, and EB: obtained funding. AG, IT, KJ, GH, ES, SB, LW, DT, EF, GG, LA, and EB: concept development and study design. AG, IT, KJ, SB, DY, and LW: data collection. GH, ES, EF, GG, LA, and EB: study coordination. AG, IT, and BE: data analysis. AG and IT: writing manuscript. AG, IT, KJ, GH, ES, GG, LA, and EB: revising manuscript. All authors read and approved the final manuscript.

## Conflict of Interest

The authors declare that the research was conducted in the absence of any commercial or financial relationships that could be construed as a potential conflict of interest.
