# Corrigendum: The PERSonalized Glucose Optimization Through Nutritional Intervention (PERSON) Study: Rationale, Design and Preliminary Screening Results

**DOI:** 10.3389/fnut.2022.836546

**Published:** 2022-02-03

**Authors:** Anouk Gijbels, Inez Trouwborst, Kelly M. Jardon, Gabby B. Hul, Els Siebelink, Suzanne M. Bowser, Dilemin Yildiz, Lisa Wanders, Balázs Erdos, Dick H. J. Thijssen, Edith J. M. Feskens, Gijs H. Goossens, Lydia A. Afman, Ellen E. Blaak

**Affiliations:** ^1^Division of Human Nutrition and Health, Wageningen University, Wageningen, Netherlands; ^2^Top Institute Food and Nutrition, Wageningen, Netherlands; ^3^Department of Human Biology, Maastricht University Medical Center+, Maastricht, Netherlands; ^4^Department of Physiology, Radboud Institute for Health Sciences, Radboud University Medical Center, Nijmegen, Netherlands; ^5^Maastricht Centre for Systems Biology, Maastricht University, Maastricht, Netherlands; ^6^Research Institute for Sport and Exercise Sciences, Liverpool John Moores University, Liverpool, United Kingdom

**Keywords:** precision nutrition, personalized nutrition, insulin resistance, metabolic phenotype, glucose homeostasis, obesity, dietary intervention study, randomized clinical trial

In the original article, there was a mistake in Supplementary Tables 3 and 4. Some numbers in these tables were incorrect. The corrected tables Supplementary Tables 3 and 4 appear below.

**Supplementary Table 3:** Overview of standardized products provided during at-home days and amounts provided per energy group.

**Meal moment (time frame)**	**Product**	**Nutrients per 100 g**					**Amounts (g) provided per energy group**			
		**Energy (kJ)**	**Fat (g)**	**Saturated fat (g)**	**Protein (g)**	**Carbohydrates (g)**	**Sugars (g)**	**6-8 MJ**	**9-11 MJ**	**12-13 MJ**
Breakfast (7am-9am)	Drink yogurt	247	0.8	0.5	3.4	8.1	7.3	400	400	400
	Gingerbread	1304	1.1	0.4	2.9	69.6	37.1	28	28	28
Snack (10am-11am)	Raisin cake	1785	21.3	6.8	6.3	51.7	35.0	60	60	60
	Banana	401	0.3	0.1	1.1	20.6	15.5	130	130	130
	Apple juice	194	0.0	0.0	0.1	11.2	10.5	200	200	200
Lunch (12am-1pm)	Wheat bread	1000	1.8	0.4	9.8	42.9	2.0	56	84	112
	Cream cheese	1540	33.3	10.7	14.0	2.7	2.0	15	30	30
	Hazelnut spread	2347	35.3	9.3	6.0	54.0	50.0	15	15	30
	Semi-skimmed milk	192	1.5	1.0	3.4	4.7	4.7	200	200	200
	Yogurt with	368	2.0	1.3	4.0	13.0	11.0	190	190	190
	strawberry sauce									
Snack (3pm-4pm)	Apple	254	0.2	0.0	0.3	13.0	10.4	135	135	135
	Potato chips	2261	33.2	5.7	6.4	52.5	1.4	28	28	28
	Lemonade	170	0.1	0.0	0.1	9.7	9.6	200	200	200
Dinner (6pm-7pm)	Macaroni meal	447	3.6	1.2	5.6	12.1	2.0	350	450	550

*Nutrient composition was calculated using the 2016 Dutch Food Composition Table^90^ kJ, kilojoule; MJ, megajoule*.

**Supplementary Table 4:** Macronutrient composition of standardized meal moments during home-days per energy group.

**Energy group**	**Meal moment (time frame)**	**Energy (kJ)**	**Fat (g)**	**Saturated fat (g)**	**Protein (g)**	**Carbohydrates (g)**	**Sugars (g)**
6-8 MJ	Breakfast (7am-9am)	1353	3.5	2.1	14.4	51.9	39.6
	Snack (10am-11am)	1980	13.2	4.2	5.4	80.2	62.2
	Lunch (12am-1pm)	2226	18.1	7.7	22.8	66.6	39.2
	Snack (3pm-4pm)	1316	9.8	1.6	2.4	51.6	33.7
	Dinner (6pm-7pm)	1565	12.7	4.2	19.7	42.2	6.9
	Total	8440	57.3	19.8	64.7	292.5	181.6
9-11 MJ	Breakfast (7am-9am)	1353	3.5	2.1	14.4	51.9	39.6
	Snack (10am-11am)	1980	13.2	4.2	5.4	80.2	62.2
	Lunch (12am-1pm)	2738	23.6	9.4	27.6	79.1	40.1
	Snack (3pm-4pm)	1316	9.8	1.6	2.4	51.6	33.7
	Dinner (6pm-7pm)	2012	16.3	5.4	25.3	54.3	8.9
	Total	9399	66.4	22.7	75.1	317.1	184.5
12-13 MJ	Breakfast (7am-9am)	1353	3.5	2.1	14.4	51.9	39.6
	Snack (10am-11am)	1980	13.2	4.2	5.4	80.2	62.2
	Lunch (12am-1pm)	3369	29.4	10.9	31.2	99.2	48.2
	Snack (3pm-4pm)	1316	9.8	1.6	2.4	51.6	33.7
	Dinner (6pm-7pm)	2459	19.9	6.6	30.9	66.3	10.9
	Total	10477	75.8	25.4	84.3	349.2	194.6

*Nutrient composition was calculated using the 2016 Dutch Food Composition Table^90^ kJ, kilojoule; MJ, megajoule*.

The authors apologize for this error and state that this does not change the scientific conclusions of the article in any way. The original article has been updated.

## Publisher's Note

All claims expressed in this article are solely those of the authors and do not necessarily represent those of their affiliated organizations, or those of the publisher, the editors and the reviewers. Any product that may be evaluated in this article, or claim that may be made by its manufacturer, is not guaranteed or endorsed by the publisher.

