# Supplementary material for: The PERSonalized Glucose Optimization Through Nutritional Intervention (PERSON) Study: Rationale, Design and Preliminary Screening Results
Source: Front Nutr. 2021 Jun 30;8:694568. doi: 10.3389/fnut.2021.694568 (PMC8278004; doi:10.3389/fnut.2021.694568)
Supplement: Supplementary file 1 [file Data_Sheet_1.PDF]

frontiers

Supplementary Material

Table S1. Inclusion and exclusion criteria for the PERSON study

**Inclusion criteria**	• Men and women aged 40-75 y
	• BMI 25-40 kg/m^2
	• Body weight stability for at least 3 months (no weight change >3kg)
	• Predominantly muscle (MIR) or liver (LIR) insulin resistant
**Exclusion criteria**	**Diseases**
	• Pre-diagnosis of type 1 or type 2 diabetes mellitus
	• Renal or hepatic malfunctioning (pre-diagnosis or determined based on alanine aminotransferase [ALAT], aspartate aminotransferase [ASAT] and creatinine values)
	• Major gastrointestinal diseases or major abdominal surgery
	• Cardiovascular diseases (e.g. heart failure) or cancer
	• High blood pressure (untreated >160/100 mmHg, drug-regulated >140/90 mmHg)
	• Diseases affecting glucose and/or lipid metabolism (e.g. pheochromocytoma, Cushing's syndrome, acromegaly)
	• Anemia defined as hemoglobin (Hb) men <8.5 and women <7.5 mmol/l
	• Diseases with a life expectation shorter than 5 years
	• Major mental disorders
	• Drug treated thyroid diseases (well-substituted hypothyroidism is allowed)
	**Medication**
	• Medication known to interfere with study outcomes (e.g. peroxisome proliferator-activated receptor-α [PPAR-α] or PPAR-γ agonists [fibrates], sulfonylureas, biguanides, α-glucosidase inhibitors, thiazolidinediones, repaglinide, nateglinide and insulin, chronic use of NSAIDs)
	• Use of anticoagulants other than acetylsalicyclic acid
	• Use of antidepressants (stable use ≥3 months prior to and during the study is allowed)
	• Use of statins (stable use ≥3 months prior to and during study allowed)
	• Use of β-blockers (only for the extensive phenotyping participants)
	• Chronic corticosteroids treatment (>7 consecutive days of treatment)
	• Use of antibiotics within 3 months prior to the study
	**Lifestyle**
	• Participation in regular sports activities (>4 hours per week)
	• Abuse of alcohol (alcohol consumption >14 units/week) and/or drugs (cannabis included)
	• Regular smoking (including use of e-cigarettes)
	**Other**
	• Pregnant or lactating women who are planning to become pregnant
	• Inability to comply with the study diet

Table S2. Ingredients and macronutrient composition of the high-fat mixed meal

	**Full-fat milk**	**Whipped cream**	**Sugar**	**Whipped ice cream**	**Total per meal**
Amount per meal (g)	125	70	5	150	350
Energy (kJ)	347.5	973.0	85	1387.5	2793.0
Protein (g)	4.5	1.5	0	5.6	11.6
Fat (g)	4.5	24.6	0	19.5	48.6
Saturated fat (g)	3.1	17.5	0	12.8	33.4
Carbohydrates (g)	5.9	2.2	5	34.5	47.5
Sugar (g)	5.9	2.2	5	31.5	44.5

Table S3. Overview of standardized products provided during at-home days and amounts provided per energy group

Meal moment (time frame)	Product	Nutrients per 100 g						Amounts (g) provided per energy group		
		Energy (kJ)	Fat (g)	Saturated fat (g)	Protein (g)	Carbohydrates (g)	Sugars (g)	6-8 MJ	9-11 MJ	12-13 MJ
Breakfast (7am-9am)	Drink yogurt	247	0.8	0.5	3.4	8.1	7.3	400	400	400
	Gingerbread	1304	1.1	0.4	2.9	69.6	37.1	28	28	28
Snack (10am-11am)	Raisin cake	1785	21.3	6.8	6.3	51.7	35.0	60	60	60
	Banana	401	0.3	0.1	1.1	20.6	15.5	130	130	130
	Apple juice	194	0.0	0.0	0.1	11.2	10.5	200	200	200
Lunch (12am-1pm)	Wheat bread	1000	1.8	0.4	9.8	42.9	2.0	56	84	112
	Cream cheese	1540	33.3	10.7	14.0	2.7	2.0	15	30	30
	Hazelnut spread	2347	35.3	9.3	6.0	54.0	50.0	15	15	30
	Semi-skimmed milk	192	1.5	1.0	3.4	4.7	4.7	200	200	200
	Yogurt with strawberry sauce	368	2.0	1.3	4.0	13.0	11.0	190	190	190
Snack (3pm-4pm)	Apple	254	0.2	0.0	0.3	13.0	10.4	135	135	135
	Potato chips	2261	33.2	5.7	6.4	52.5	1.4	28	28	28
	Lemonade	170	0.1	0.0	0.1	9.7	9.6	200	200	200
Dinner (6pm-7pm)	Macaroni meal	447	3.6	1.2	5.6	12.1	2.0	350	450	550

Nutrient composition was calculated using the 2016 Dutch Food Composition Table[90]
kJ, kilojoule; MJ, megajoule

Table S4. Macronutrient composition of standardized meal moments during home-days per energy group

Energy group	Meal moment (time frame)	Energy (kJ)	Fat (g)	Saturated fat (g)	Protein (g)	Carbohydrates (g)	Sugars (g)
6-8 MJ	Breakfast (7am-9am)	1353	3.5	2.1	14.4	51.9	39.6
	Snack (10am-11am)	1980	13.2	4.2	5.4	80.2	62.2
	Lunch (12am-1pm)	2226	18.1	7.7	22.8	66.6	39.2
	Snack (3pm-4pm)	1316	9.8	1.6	2.4	51.6	33.7
	Dinner (6pm-7pm)	1565	12.7	4.2	19.7	42.2	6.9
	Total	8440	57.3	19.8	64.7	292.5	181.6
9-11 MJ	Breakfast (7am-9am)	1353	3.5	2.1	14.4	51.9	39.6
	Snack (10am-11am)	1980	13.2	4.2	5.4	80.2	62.2
	Lunch (12am-1pm)	2738	23.6	9.4	27.6	79.1	40.1
	Snack (3pm-4pm)	1316	9.8	1.6	2.4	51.6	33.7
	Dinner (6pm-7pm)	2012	16.3	5.4	25.3	54.3	8.9
	Total	9399	66.4	22.7	75.1	317.1	184.5
12-13 MJ	Breakfast (7am-9am)	1353	3.5	2.1	14.4	51.9	39.6
	Snack (10am-11am)	1980	13.2	4.2	5.4	80.2	62.2
	Lunch (12am-1pm)	3369	29.4	10.9	31.2	99.2	48.2
	Snack (3pm-4pm)	1316	9.8	1.6	2.4	51.6	33.7
	Dinner (6pm-7pm)	2459	19.9	6.6	30.9	66.3	10.9
	Total	10477	75.8	25.4	84.3	349.2	194.6

Nutrient composition was calculated using the 2016 Dutch Food Composition Table[90]
kJ, kilojoule; MJ, megajoule

Table S5. Glucose homeostasis parameters derived from OGTT according to insulin resistance phenotype

	**No MIR/LIR (n=227)**	**MIR (n=121)**	**LIR (n=61)**	**Combined MIR/LIR (n=156)**	***P*-value**
Fasting glucose (mmol/L)	5.5 (5.4, 5.6)	5.4 (5.3, 5.5)	5.6 (5.4, 5.7)	5.6 (5.5, 5.7)†	0.005
Fasting insulin (pmol/L)	38.7 (36.6, 41.0)	51.6 (47.8, 55.8)§	56.1 (50.4, 62.6)§	89.3 (83.4, 95.6)§†‡	<0.001
2-hr glucose (mmol/L)	5.9 (5.7, 6.2)	6.9 (6.5, 7.3)§‡	5.9 (5.5, 6.4)†	7.0 (6.6, 7.3)§‡	<0.001
2-hr insulin (pmol/L)	220.6 (200.3, 242.9)	488.8 (428.0, 557.9)§‡	316.3 (262.7, 381.0)§†	759.6 (676.2, 853.7)§†‡	<0.001
iAUC glucose (AU)	203 (187, 219)	242 (217, 270)	255 (219, 297)	285 (259, 314)§	<0.001
iAUC insulin (AU)	27612 (25918, 29409)	47512 (43522, 51822)§	56855 (50334, 64236)§	92554 (85757, 99915)§†‡	<0.001
HOMA-IR (AU)	1.4 (1.3, 1.5)	1.8 (1.6, 1.9)§	2.0 (1.8, 2.3)§	3.2 (3.0, 3.5)§†‡	<0.001
HOMA-β (AU)	58.0 (54.9, 61.2)	82.1 (76.2, 88.6)§	79.7 (71.7, 88.7)§	124.2 (116.2, 132.7)§†‡	<0.001
Matsuda index (AU)	16.7 (15.7, 17.8)	10.8 (9.9, 11.8)§	9.6 (8.5, 10.9)§	5.7 (5.3, 6.2)§†‡	<0.001
Disposition index (AU)	362 (336, 390)	331 (299, 367)‡	465 (403, 537)§†	350 (320, 383))‡	0.002
MISI (AU)	0.217 (0.202, 0.232)	0.060 (0.055, 0.067)§‡	0.148 (0.129, 0.170)§†	0.049 (0.045, 0.054)§†‡	<0.001
HIRI (AU)	253 (240, 266)	334 (312, 359)§‡	712 (645, 787)§†	840 (789, 894)§†‡	<0.001

Differences between tissue-specific IR groups were assessed using ANCOVA with adjustment for sex and Bonferroni post-hoc pairwise comparisons. Data were logtransformed to improve normality and reported as adjusted geometric means with 95% confidence interval.
§ significantly different from No MIR/LIR (p<0.05)
† significantly different from MIR (p<0.05)
‡ significantly different from LIR (p<0.05)
OGTT, oral glucose tolerance test; iAUC, incremental area under the curve; AU, arbitrary units; HOMA-IR, homeostasis model assessment of insulin resistance; HOMA- β, homeostasis model assessment of β-cell function; MISI, muscle insulin sensitivity; HIRI, hepatic insulin resistance.
